# Eu^3+^ doped ZnAl layered double hydroxides as calibrationless, fluorescent sensors for carbonate†

**DOI:** 10.1039/d3cc03066k

**Published:** 2023-10-30

**Authors:** Alysson F. Morais, Ivan G. N. Silva, Bruno J. Ferreira, Alexandre C. Teixeira, Sreeprasanth P. Sree, Huayna Terraschke, Fernando A. Garcia, Eric Breynaert, Danilo Mustafa

**Affiliations:** a Instituto de Física da Universidade de São Paulo 05508-090 – São Paulo SP Brazil dmustafa@if.usp.br; b Center for Surface Chemistry and Catalysis KU Leuven B-3001 Leuven Belgium alysson.morais@kuleuven.be; c Department of Materials Engineering KU Leuven 3001 Leuven Belgium; d Institut für Anorganische Chemie, CAU Kiel 24118 Kiel Germany; e NMR/X-Ray platform for Convergence Research (NMRCoRe) KU Leuven 3001 Leuven Belgium

## Abstract

The photoluminescence properties (PL) of Eu^3+^ hosted in the hydroxide layers of layered double hydroxides (LDHs) enables calibrationless quantification of anions in the interlayers. The concept is demonstrated during the nitrate-to-carbonate ion exchange in Zn^2+^/Al^3+^/Eu^3+^ LDHs and can be implemented as a remote optical sensor to detect intrusion of anions such as Cl^−^ or CO_3_^2−^.

## Abstract 2

*In situ* remote sensing of carbonate anions is enabled by incorporation of lanthanides in layered double hydroxides (LDHs). Quantification of carbonate immobilized in LDHs is here illustrated by following the visible luminescence of Eu^3+^-doped LDHs.

## Graphical abstract

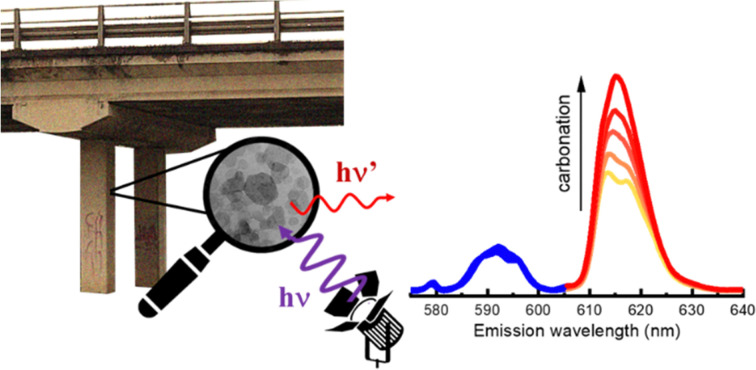

Corrosion of concrete rebar is one of the dominant mechanisms limiting the lifetime of reinforced concrete constructions.^1,2^ Fresh concrete contains a mixture of highly alkaline materials, ensuring a high pH concrete pore water (pH 12–13) environment. This high pH induces the formation of a passivating layer on the surface of the steel rebar, thus reducing corrosion. Carbonation of concrete, *i.e.*, the uptake of carbon dioxide from the air, can cause the passivating layer in steel rebars to shrink. In this mechanism, CO_2_ reacts with the hydroxides in the concrete, converting them into metal carbonates and decreasing the local pH. When the low pH front reaches the steel rebar and the steel is depassivated, corrosion kicks thus jeopardising the lifetime of the construction.^3–6^ Aside its impact in the rebar passivation layer, carbonation also increases the sensitivity of concrete to other corrosion mechanisms such as chloride attack, and thus is a key determinant of the service life of concrete structures.^7^

The progress of carbonation fronts in concrete structures is typically visualised by spraying a phenolphthalein solution on concrete cores freshly drilled out of the concrete cover. In contact with pristine concrete, phenolphthalein turns bright pink while its colour fades or completely disappears when carbonation has occurred, thus indicating the pH has been lowered below pH 10.^8,9^ This procedure is, however, detrimental to the concrete structure. For critical infrastructure, *e.g.*, bridges, dams, and nuclear containments, a reliable, *in situ*, non-destructive method to characterize the evolution of the carbonation front as a function of the distance to the rebar would be advantageous, allowing improved planning of maintenance actions. A sensor allowing non-destructive remote detection of the progress of the carbonation front could be created from lanthanide-doped LDHs combined with fibre-based detection of their PL.

Layered double hydroxides are materials formed by the stacking of positively charged 2D networks (or layers) of edge-sharing octahedra of divalent and trivalent metal cations bridged by hydroxyl groups. The stacking of these layers is mediated by anionic species intercalated between the hydroxide layers.^10–14^ As result of the high variability in cation and anion composition, LDHs are promising materials for use as anion exchangers, adsorbents, catalysts, for controlled release of pharmaceutical components, and as solid electrolytes for battery applications.^15–22^ Recently, even nanotubular, luminescent, and mixed ligand luminescent LDHs have been reported.^10,23–25^ Introduction of trivalent europium (Eu^3+^) in the hydroxide layers of LDHs produces luminescent LDH phases whose PL depend on the anionic spacer.^25–27^ At the same time, the anions in LDHs are exchangeable based on their affinity to the LDH host, carbonate (CO_3_^2−^) standing high in the LDH affinity series.^28–30^ The combination of high affinity to carbonate and the anion dependent PL of LDHs can then turn these materials into a class of high affinity, calibrationless carbonate sensors with potential application for the *in situ*, non-destructive assessment of the evolution of the carbonation front in reinforced concrete.

Here we investigate and report on the structural and luminescent properties of Eu-doped Zn^2+^/Al^3+^ LDHs. The changes in the PL of these LDHs have been followed in the equilibrium state and *in situ* during anion exchange reactions where pristine LDHs initially intercalated with nitrate (LDH-NO_3_^−^) were dispersed in sodium carbonate solutions at different concentrations. These anion exchange reactions led to LDHs where nitrate was completely or partially exchanged for carbonate, which could be qualitatively and quantitatively assessed by, respectively, powder X-ray diffraction (PXRD) and elemental analysis. LDHs with remarkably different luminescence properties have been obtained after the anion exchange reactions, from which the local changes in the Eu^3+^ coordination could be inferred. Extended X-ray absorption fine structure (EXAFS^31^) spectroscopy shows Eu^3+^ is hosted in the hydroxide layers of the LDHs, in positions normally occupied by Al^3+^. Further, insights on the coordination changes caused by anion exchange were given by EXAFS data analysis.

In brief, the inclusion of Eu^3+^ in the hydroxide layers of LDHs demands no special or modified synthetic routes as either Eu(NO_3_)_3_ or EuCl_3_, salts highly soluble in water, can readily serve as precursors for Eu^3+^ in synthetic procedures involving controlled hydrolysis.^10,32^ By this strategy, Eu^3+^ can be doped in the hydroxide layers of Zn/Al LDHs up to Eu/(Eu + Al) ratios of about 15% without segregation of Eu^3+^ in additional crystalline phases.^25^ Further, analysis of Eu L^III^-edge EXAFS data confirms the successful incorporation of Eu^3+^ in the hydroxide layers of these LDHs in replacement of Al^3+^.^25–27^ For the present work, nitrate intercalated Eu^3+^-doped Zn^2+^/Al^3+^ LDHs (LDH-NO_3_^−^) were prepared by coprecipitation of Zn^2+^, Al^3+^ and Eu^3+^ at constant pH (see ESI†). This procedure successfully yields pristine NO_3_^−^-intercalated LDHs with a characteristic basal distance of 8.6 Å, as revealed by the average between the interplanar distances calculated for the (003) and (006) Bragg reflections observed at, respectively, 10.4° and 20.4° 2*θ* in their PXRD pattern (Fig. 1).^33^

**Fig. 1:**
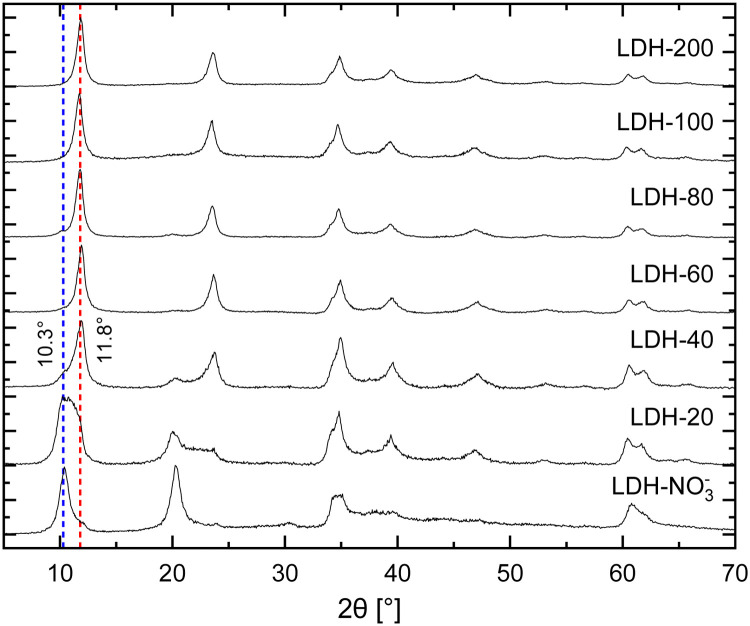
PXRD patterns of pristine LDH-NO_3_^−^ and the anion exchanged samples LDH-*A* (*A* = 20, 40, 60, 80, 100 and 200 standing for the initial carbonate concentration in mmol L^−1^ in the anion exchange solution). The NO_3_^−^- and CO_3_^2−^-intercalated phases can be identified by the characteristic basal reflections at 10.3 and 11.8° 2*θ*, respectively.

Carbonate sequestration with release of nitrate when pristine LDH-NO_3_^−^ is dispersed in a Na_2_CO_3_ solution is evident both from elemental analysis (Table S1, ESI†) and PXRD (Fig. 1). CO_3_^2−^-intercalated LDHs feature a shorter basal distance as compared to LDH-NO^3−^, making a basal reflection to appear at 11.8° 2*θ* for the CO_3_^2−^-intercalated LDHs.^14,34^

Table S1 (ESI†) shows the elemental composition of LDH-NO_3_^−^ before and after anion exchange. Nitrate was exchanged for carbonate by dispersing LDH-NO_3_^−^ in solutions of different initial carbonate concentrations (samples dubbed LDH-*A*, *A* = 20, 40, 60, 80, 100 and 200 standing for the initial carbonate concentration, in mmol L^−1^, in the anion exchange solution). More details on the anion exchange procedure are provided in the ESI.† In line with PXRD, the carbon content in the samples increases with the increase in the CO_3_^2−^ concentration in the exchange solution, confirming that LDHs are effective materials for carbonate uptake. At the same time, the nitrogen content decreases, showing that carbonate is fixed in the samples at the expense of the nitrate anions. The effectiveness of the anion exchange in the samples can be experimentally quantified by the equivalent fraction *X* = (1 + 0.5 × mol% NO_3_^−^/mol% CO_3_^−^)^−1^, which defines the LDH composition as in the general formula [Zn_2_Al_0.95_Eu_0.05_(OH)_6_][(NO_3_^−^)_1−*X*_(CO_3_^2−^)_*X*/2_]·*m*H_2_O. From the point of view of elemental analysis, carbonate saturation in the LDHs is reached for initial CO_3_^2−^ concentrations between 80 (*X* = 0.95) and 100 mmol L^−1^ (*X* = 1.0). Interestingly, as seen from PXRD, CO_3_^2−^ already dominates the interlayer space of the LDHs for concentrations above 60 mmol L^−1^. Above this concentration, the structural properties of the LDHs hinder any tentative to discriminate the presence or absence of nitrate by using PXRD.

The PL spectra obtained for LDH-*A* (*A* = 20, 40, 60, 80, 100 and 200) are shown on Fig. 2A. In the spectral region from 580 to 710 nm, the spectra show the emission bands of Eu^3+^, indicated by the term symbols of its 4f electronic states. Eu^3+^ is a lanthanide element and, as such, features a set of narrow emission bands arising from its 4f–4f electronic transitions.^35^ Appearing in the spectra shown on Fig. 2A are the ^5^D_0_ → ^7^F_1_ and ^5^D_0_ → ^7^F_1_ transitions. While the intensity *I*(^5^D_0_ → ^7^F_1_) is known to be largely independent on the host Eu^3+^ is embedded in, the intensity *I*(^5^D_0_ → ^7^F_2_) is very sensitive to the geometry and chemical environment around the luminescent centre.^35–37^ This makes *I*(^5^D_0_ → ^7^F_1_) an internal luminescence standard useful when comparing luminescence spectra of different Eu-containing samples, turning Eu-containing LDH into calibrationless molecular sensors.

**Fig. 2:**
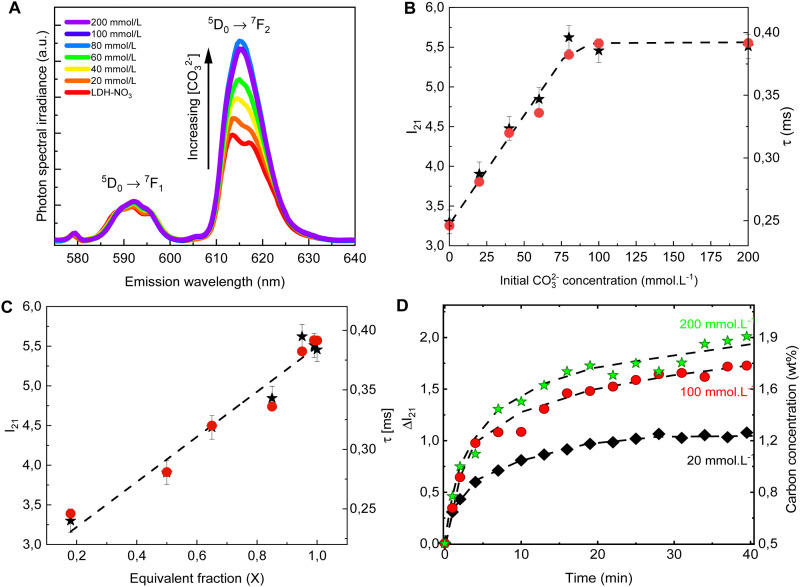
Emission spectra (A), *I*_21_ = *I*(^5^D_0_ → ^7^F_2_)/*I*(^5^D_0_ → ^7^F_1_) intensity ratio (B, ★, left scale) and luminescence decay lifetime (B, ●, right scale) of the anion exchanged LDHs after equilibration at different concentrations of CO_3_^2−^. The spectra are normalized by the integral intensity of the (Eu^3+^)^5^D_0_ → ^7^F_1_ emission band. (C) *I*_21_ intensity ratio (★, left scale) and luminescence decay lifetime (●, right scale) as a function of the carbonate equivalent fraction (*X*) in the LDH samples. (D) Changes in the *I*_21_ intensity ratio over time in the *in situ* experiments with different initial carbonate concentrations. The right scale shows the carbon concentration calculated from eqn (1). The dashed lines are guides to the eye.

In the PL spectra shown on Fig. 2A, all spectral profiles have been normalized by the integral intensity *I*(^5^D_0_ → ^7^F_1_). With this internal normalization, the (Eu^3+^)^5^D_0_ → ^7^F_2_ emission has been observed to increase (Fig. 2B and C) with the CO_3_^2−^ concentration in the anion exchange solution. While the PXRD patterns of LDH-60 and LDH-80 are very similar, the *I*_21_ = *I*(^5^D_0_ → ^7^F_2_)/*I*(^5^D_0_ → ^7^F_1_) intensity ratio of these two samples are strikingly different, as seen from Fig. 2B, thus showcasing the sensitivity of the luminescence properties of Eu-containing LDH to determine differences in the carbonate content between two different LDH hosts. Also the decay lifetime (Fig. 2B. C and Table S2, Fig. S1, ESI†) of the (Eu^3+^)^5^D_0_ → ^7^F_2_ transition has been observed to increase with the uptake of carbonate from the anion exchange solution. With carbonate concentrations exceeding 100 mmol L^−1^ these changes cease, indicating the saturation of the anion sites in the LDHs, which is consistent with the stoichiometry derived from elemental analysis (Table S1 and Fig. S2, ESI†).

Both the luminescence decay lifetime and the *I*(^5^D_0_ → ^7^F_2_)/*I*(^5^D_0_ → ^7^F_1_) intensity ratio in the Eu-containing LDHs strongly correlate with the CO_3_^2−^ load in the samples, as shown in Fig. 2C and Fig. S2 (ESI†). The *I*(^5^D_0_ → ^7^F_2_)/*I*(^5^D_0_ → ^7^F_1_) intensity ratio is shown to feature a linear relationship with the CO_3_^2−^ concentration in the interlayer gallery (Fig. S2, ESI†). This relationship can then be extrapolated for an ideal LDH phase containing only NO_3_^−^ (no CO_3_^2−^), giving the value: *I*(^5^D_0_ → ^7^F_2_)/*I*(^5^D_0_ → ^7^F_1_) = 2.8 ± 0.7, from which the CO_3_^2−^ loading in the LDHs can be written as:

1 wt% *C* = 0.75 × (*I*_21_ − 2.8),

where *I*_21_ is the experimental *I*(^5^D_0_ → ^7^F_2_)/*I*(^5^D_0_ → ^7^F_1_) intensity ratio. This correlation can then be used for *in situ* measurements of the carbonate loading in the LDHs.

To follow the PL of LDHs *in situ* during a CO_3_^2−^ uptake experiment, a setup comprising a portable fluorimeter equipped with an optical fibre to monitor the emission of the sample was assembled. A commercial 10 W UV LED lamp (Fig. S3, ESI†) was used as excitation source. Carbonate solutions with three different concentrations were added to three LDH suspensions, with the LDHs used in the different suspensions originated from the same batch. For these *in situ* experiments, the initial carbonate concentrations were chosen to be well below (20 mmol L^−1^), similar to (100 mmol L^−1^) and well beyond (200 mmol L^−1^) the concentration seen by elemental analysis to cause saturation of the anionic sites in the LDHs. As evident from Fig. 2D, the carbonate uptake by the LDHs is followed by an increase in time of the *I*(^5^D_0_ → ^7^F_2_)/*I*(^5^D_0_ → ^7^F_1_) intensity ratio. Eqn (1) was used to calculate the carbonate concentration in the solid phase based on the measured PL. The carbonate load in the solid phase linearly increases in time in the initial steps of the exchange reaction. Then, as equilibrium is reached, a plateau starts to form, with the stabilization of the carbonate loading in the solid phase. Similar carbonate uptake profiles are observed for the LDHs dispersed in the solutions with initial carbonate concentration of 100 and 200 mmol L^−1^, again a consequence of the saturation of the LDHs.

As the PL data of Eu-containing compounds is sensitive to the presence of water and to the coordination geometry around the luminescent activator, it is clear from Fig. 2A that the local environment around europium in the LDHs is changed when nitrate is exchanged for carbonate. To characterize these changes, the Eu L^III^ EXAFS spectra of LDH-NO_3_ and a carbonate-intercalated LDH-CO_3_ were fitted and analysed together with the PL data (see Fig. 3 and Fig. S4, Table S3, ESI†). A detailed description of the EXAFS fitting procedure is available in the ESI.† 6 Zinc atoms are found at 4.00 and 3.93 Å from Eu in LDH-NO_3_ and LDH-CO_3_, respectively, in full accordance with Eu^3+^ being hosted in the hydroxide layers of the LDHs, replacing Al^3+^. In both samples, the first coordination shell around Eu comprises 7 oxygen atoms at 2.42 Å, with an additional oxygen appearing at 3.03 Å in LDH-NO_3_ and at a slightly shorter distance, at 2.85 Å, in LDH-CO_3_. For LDH-NO_3_, this additional oxygen coordination is likely to belong to coordination water, as already reported elsewhere.^25–27^ For LDH-CO_3_, a different origin is more likely, based on the much longer (Eu^3+^)^5^D_0_ → ^7^F_2_ luminescence decay lifetime (*τ*) observed for this sample as compared to LDH-NO_3_ (see Fig. 2B). The (Eu^3+^)^5^D_0_ → ^7^F_2_ luminescence decay lifetime in Eu-containing compounds is strongly dependent on the number of OH quenchers around the Eu^3+^ activators, the lifetime increasing when OH quenchers are removed from the first coordination shell of Eu^3+^.^38–40^ The increase in τ as observed in Fig. 2B and C would then be consistent with the replacement of the coordination water around Eu upon carbonate adsorption.

**Fig. 3:**
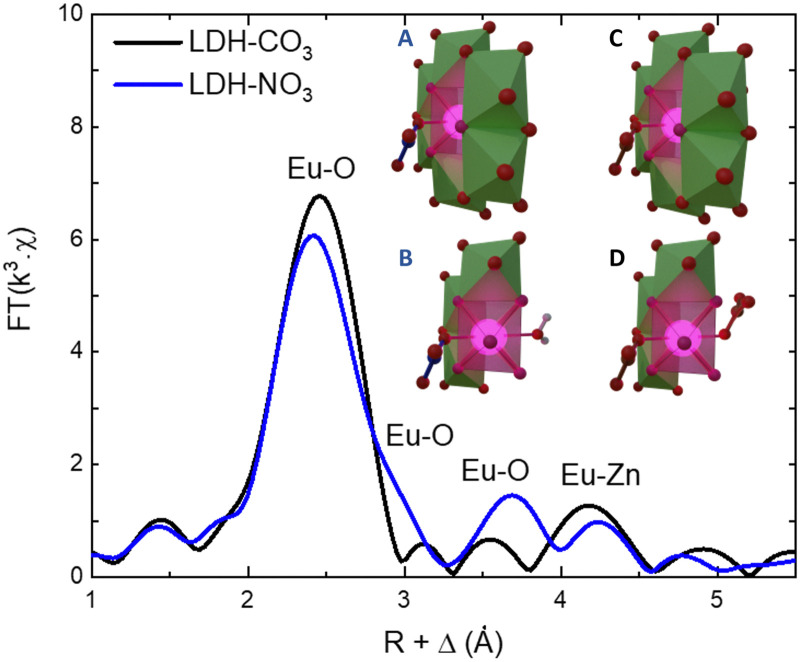
Phase-corrected Fourier transform of the Eu L^III^-edge EXAFS data for LDH-NO_3_ and a carbonate-intercalated Zn^2+^/Al^3+^/Eu^3+^ LDH (LDH-CO_3_). The inset illustrates the coordination structure of Eu^3+^ in LDH-NO_3_ (A) and (B) and LDH-CO_3_ (C) and (D). For better visualization of the Eu^3+^ coordination with the interlayers, B and D present a cut in the structure where some Zn-centred polyhedrons have been supressed. Europium is coordinated to 8 oxygen atoms, of which 6 are attributed to OH groups in the hydroxide layers. In LDH-NO_3_ one additional coordinated oxygen come from interlayer water and another from interlayer nitrate (B), whereas in LDH-CO_3_ the 2 additional coordinated oxygen come from CO_3_^2−^ anions (D).^25–27^

In summary, the here reported experiments show that the introduction of Eu^3+^ in the hydroxide layers of LDHs enables the optical assessment of the degree of carbonate uptake by the LDH matrix. Since the intensity of the magnetic dipole transition (Eu^3+^)^5^D_0_ → ^7^F_1_ is largely independent on the host, it can serve as an internal standard during the analysis of PL spectra, turning Eu^3+^-doped LDHs into calibrationless carbonate sensors with potential application for *in situ*, non-destructive assessment of the progress of the carbonation front in concrete. This would enable continuous, remote quality control of critical infrastructure built from reinforced concrete.

This research was funded by Fundação de Amparo à Pesquisa do Estado de São Paulo (FAPESP, 2015/19210-0, 2022/01314-8, 2019/25665-1 and 2018/13837-0) and Coordenação de Aperfeiçoamento de Pessoal de Nível Superior (CAPES, 1723707 and 88887.371434/2019-00). The authors acknowledge CNPEM-LNLS for concession of beamtime (Proposals No. 20190148 and 20180133). E. B. and A. F. M. acknowledge support from the European Research Council through an Advanced Research Grant under the European Union's Horizon 2020 research and innovation programme (No. 834134 WATUSO). A. F. M. acknowledges support from the European Union’s Horizon Europe programme through a Marie Skłodowska-Curie postdoctoral fellowship (No. 101063656, H2E). Christine E. A. Kirschhock is kindly acknowledged for the illustration in the inset of Fig. 3. NMRCoRe acknowledges the Flemish government, department EWI for financial support as International Research Infrastructure (I001321N: Nuclear Magnetic Resonance Spectroscopy Platform for Molecular Water Research).

## Conflicts of interest

There are no conflicts to declare.

## Supplementary Material

CC-059-D3CC03066K-s001
