# Supplementary material for: Eu3+ doped ZnAl layered double hydroxides as calibrationless, fluorescent sensors for carbonate
Source: Chem Commun (Camb). 2023 Oct 30;59(91):13571–4. doi: 10.1039/d3cc03066k (PMC10644987; doi:10.1039/d3cc03066k)
Supplement: CC-059-D3CC03066K-s001 [file CC-059-D3CC03066K-s001.pdf]

Electronic Supplementary Material (ESI) for Chemical Communications.
This journal is © The Royal Society of Chemistry 2023

Electronic Supplementary Information (ESI) – ChemComm D3CC03066K

Eu^{3+} doped ZnAl layered double hydroxides as calibrationless, fluorescent sensors for carbonate

Alysson F. Morais[a,b,e,*], Ivan G.N. Silva[a], Bruno J. Ferreira[a], Alexandre C. Teixeira[a], Sreeprasanth P. Sree[b,c], Huayna Terraschke[d], Fernando A. Garcia[a], Eric Breynaert[b,e] and Danilo Mustafa[a,*]

[a] Instituto de Física da Universidade de São Paulo, 05508-090 – São Paulo, SP, Brazil, dmustafa@if.usp.br

[b] Center for Surface Chemistry and Catalysis, KU Leuven, 3001, Leuven, Belgium, alysson.morais@kuleuven.be

[c] Department of Materials Engineering, KU Leuven, 3001, Leuven, Belgium

[d] Institut für Anorganische Chemie, CAU Kiel, 24118, Kiel, Germany

[e] NMR/X-Ray platform for Convergence Research (NMRCoRe), KU Leuven, 3001, Leuven, Belgium

Experimental

Synthesis of materials: Eu^{3+}-doped Zn^{2+}/Al^{3+} layered double hydroxides intercalated with nitrate (LDH-NO_3^-) were prepared by coprecipitation of Zn^{2+}, Al^{3+} and Eu^{3+} at constant pH. For the synthesis, a ten-millilitres aqueous solution (solution A) containing $Zn(NO_3)_2$, $Al(NO_3)_3$ and $Eu(NO_3)_3$ at concentrations of 0.66, 0.314 and 0.016 mol.L^{-1}, respectively, was added dropwise (10 mL.h^{-1}) into 200 mL of Milli-Q water (solution B). During the dosing, the pH of solution B was kept in the range 7.8-8.2 by the addition of a 1 mol.L^{-1} NaOH solution by the automatic titrator Metrohm 785 DMP. After the end of the synthesis, the resulting slurry was aged statically for two days at 60 °C to optimize crystallization and subsequently centrifuged, rinsed with distilled water and dried in a drying oven at 60 °C for 3 days. The final solid was ground with mortar and pestle to produce a fine powder. For the synthesis of LDH-CO_3^{2-}, the same procedure was repeated, but solution B comprised an aqueous solution of Na_2CO_3 0.5 mol.L^{-1}.

Anion exchange: To exchange the nitrate originally intercalated in the LDHs, 300 mg of pristine LDH-NO_3 were dispersed by continuous stirring in 5 mL of a Na_2CO_3 aqueous solutions at concentrations of 20, 40, 60, 80, 100 and 200 mmol.L^{-1} (samples LDH-A, A = 20, 40, 60, 80, 100 and 200). The suspensions were equilibrated for 24 h and then centrifuged and washed with deionized water up to a final dilution of more than 100 times. After the final centrifugation step the product was dried at 60 °C for 3 days.

Characterization: Powder X-ray diffraction (PXRD) was performed in a Bruker D8 (Massachusetts, EUA) diffractometer (CuKα, 1.5418 Å). For Carbon and Nitrogen (CHN) elemental analysis, a Perkin Elmer (Massachusetts, EUA) 2400 series ii elemental analyzer was used. For Zn, Al and Eu quantification, Inductively Coupled Plasma Optical Emission Spectrometry (ICP-OES) was performed with a Spectro Arcos (SPECTRO Analytical Instruments GmbH, Germany) optical spectrometer with radial view. The *ex situ* photoluminescence measurements were performed in a FS5 Spectrofluorometer (Edinburgh Instruments) spectrofluorometer with a 450 W Xenon lamp equipped with singular monochromators both at the excitation and the emission optics. All spectra shown were corrected for the intensity profile of the Xenon lamp and detection response. X-ray absorption spectroscopy (XAS) experiments were performed at the XAFS2[1] beamline of the Brazilian Synchrotron Light Laboratory (LNLS, Campinas, Brazil). X-ray absorption near edge structure (XANES) and extended X-ray absorption fine structure (EXAFS) were collected at the Eu L^{III}-edge (6.9769 keV). A double-crystal Si(111) monochromator was used. The powder samples were pressed into pellets and the absorption spectra were measured in the fluorescence mode.

In situ photoluminescence measurements: For the in situ measurements of the luminescence properties of LDHs during anion exchange reactions, 300 mg of pristine LDH-NO_3 were suspended in 5 mL of a Na_2CO_3 aqueous solutions at concentrations of 20, 100 and 200 mmol.L^{-1}. The LDHs used in the different suspensions originated from the same batch. To follow the luminescence properties of LDHs *in situ* during the anion exchange reaction, a setup comprising an Ocean Optics USB2000 portable fluorimeter equipped with an optical fibre to monitor the emission of the sample was assembled. A commercial 10 W UV LED lamp (**Figure S2**) was used as excitation source and was place on top of the suspension.

Results

Table S1. Experimental chemical composition of pristine LDH-NO_3 and LDH-A (A = 20, 40, 60, 80, 100 and 200) as derived from elemental analysis.

Sample	Zn wt%	Al wt%	Eu wt%	C wt%	N wt%	Empirical formula*
**ZnAlEu-NO_3^-**	36.4	7.66	1.82	0.34	3.42	$[Zn_{1.9}Al_{0.96}Eu_{0.040}(OH)_{5.8}][(NO_3^-)_{0.82}(CO_3^{2-})_{0.09}]\cdot nH_2O$
**LDH-20**	36.3	7.56	1.60	0.95	2.20	$[Zn_{1.9}Al_{0.96}Eu_{0.036}(OH)_{5.8}][(NO_3^-)_{0.54}(CO_3^{2-})_{0.27}]\cdot nH_2O$
**LDH-40**	37.6	7.91	1.76	1.22	1.50	$[Zn_{1.9}Al_{0.96}Eu_{0.038}(OH)_{5.8}][(NO_3^-)_{0.35}(CO_3^{2-})_{0.33}]\cdot nH_2O$
**LDH-60**	39.2	8.45	1.99	1.68	0.25	$[Zn_{1.9}Al_{0.96}Eu_{0.040}(OH)_{5.7}][(NO_3^-)_{0.05}(CO_3^{2-})_{0.43}]\cdot nH_2O$
**LDH-80**	39.0	8.16	1.88	1.86	0.18	$[Zn_{1.9}Al_{0.96}Eu_{0.039}(OH)_{5.8}][(NO_3^-)_{0.04}(CO_3^{2-})_{0.49}]\cdot nH_2O$
**LDH-100**	39.4	8.28	1.86	2.20	0.005	$[Zn_{1.9}Al_{0.96}Eu_{0.038}(OH)_{5.8}][(CO_3^{2-})_{0.57}]\cdot nH_2O$
**LDH-200**	39.2	8.36	1.89	2.02	0.055	$[Zn_{1.9}Al_{0.96}Eu_{0.039}(OH)_{5.7}][(NO_3^-)_{0.01}(CO_3^{2-})_{0.52}]\cdot nH_2O$

* The NO_3^- stoichiometry in LDH-100 is lower than 0.002 and has not been shown.

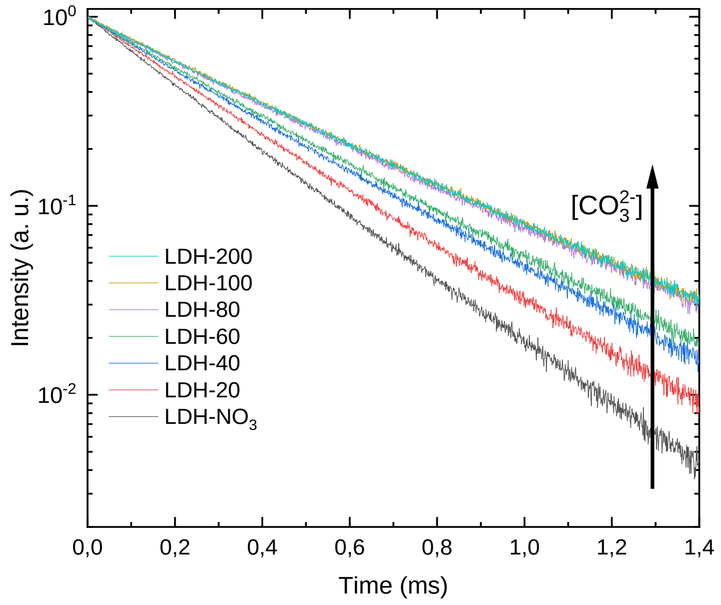

Figure S1. Luminescence decay curves monitored on the $(Eu^{3+})^5D_0 \rightarrow {}^7F_2$ emission band after direct excitation of the Eu^{3+} activators (394 nm).

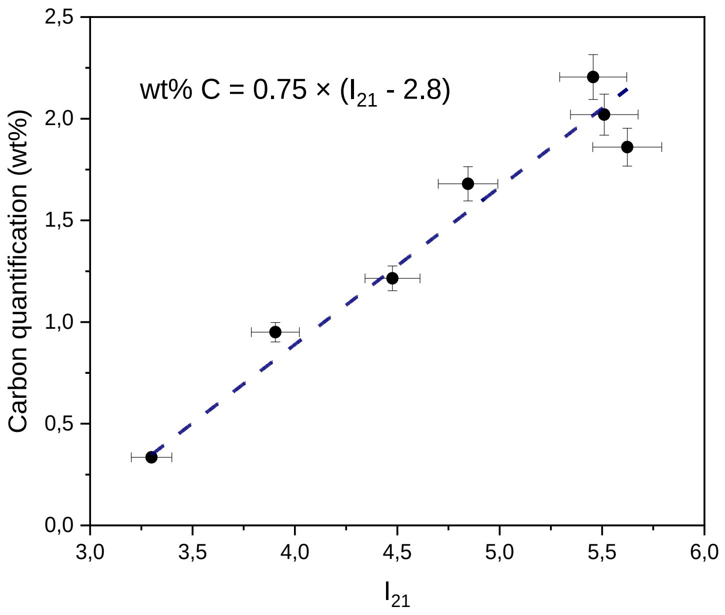

Figure S2. Calibration data for carbon quantification in the LDHs as a function of the $I_{21} = I(^5D_0 \rightarrow {}^7F_2)/ I(^5D_0 \rightarrow {}^7F_1)$ luminescence ratio.

Table S2. Luminescence decay lifetime of the $(Eu^{3+})^5D_0 \rightarrow {}^7F_1$ transition in the LDH samples after direct excitation of Eu^{3+} at 394 nm.

**Sample**	**Lifetime (ms)**	**Goodness of the fit (R-squared)**
LDH-NO_3	0.246(3)	0.99984
LDH-20	0.281(3)	0.99977
LDH-40	0.320(3)	0.99970
LDH-60	0.336(3)	0.99971
LDH-80	0.382(3)	0.99959
LDH-100	0.391(3)	0.99997
LDH-200	0.392(3)	0.99966

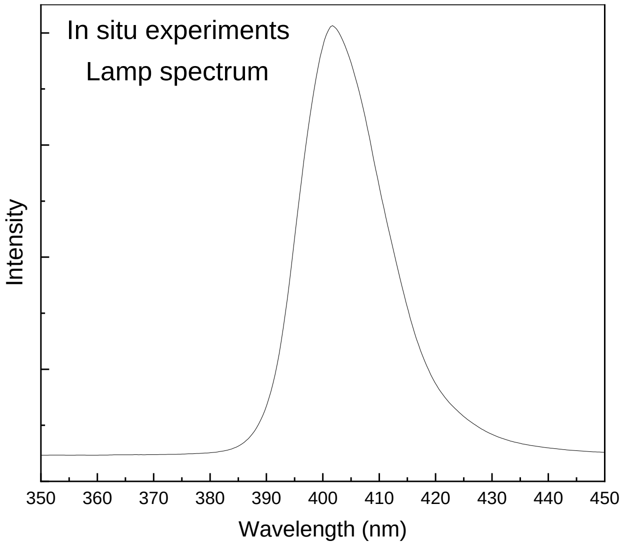

Figure S3. Spectrum of the UV lamp used as excitation source for the *in situ* PL measurements.

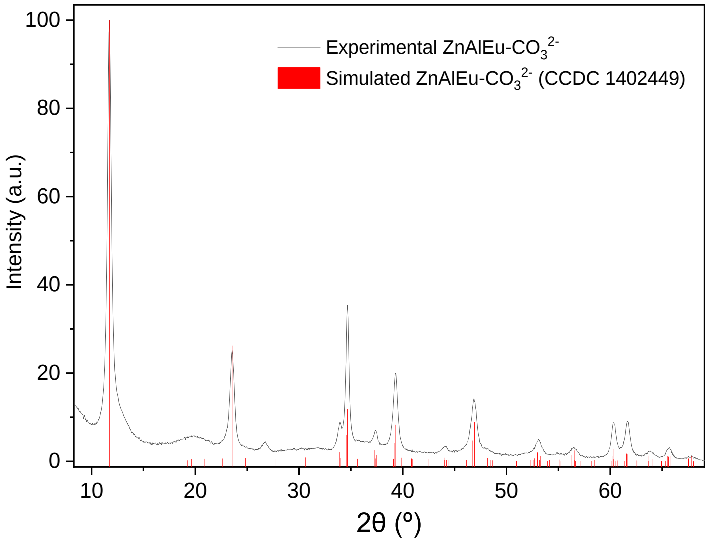

Figure S3. PXRD pattern of LDH-CO_3^{2-} and comparison to a reference structure (CCDC 1402449) of a ZnAl LDH intercalated with carbonate as shown on Ref.[2].

EXFAS data analysis: For the EXAFS data analysis, an amplitude reduction factor $S_0^2 = 0.95 \pm 0.11$ has been obtained from ab-initio calculations[3] using the FEFF8.4 code[4] for the model compound Eu_2O_3 (cubic phase[5], coordination number = 6, see Ref. [6]). Chemical transferability has been assumed and this amplitude factor was used in the analysis of the LDH samples. The Demeter platform[7] has been used to model and fit the EXAFS data. Crystallographic information from layered Europium hydroxides[8], assumed to be crystal analogs for the local environment of Eu^{3+} in the LDHs, were used as input for the calculations implemented along with IFEFFIT. The coordination of Eu^{3+} in LDH-NO_3 and LDH-CO_3 as derived from the fitting of the Eu^{3+} L^{III}-edge EXAFS data (**Figure S4**) is summarized on **Table S3**. Detailed inspection of the Fourier transformed EXAFS spectra for each coordination shell observed in the spectra revealed no evidence of high-Z backscatterers in the near coordination environment of Eu^{3+}, excluding the presence of Eu segregation in Eu-rich side phases in both samples. In LDH-NO_3, Europium is surrounded by 7 Oxygen atoms at 2.42 Å, 1 Oxygen atom at 3.03 Å, 4 Oxygen atom at 3.57 Å and 6 Zn atoms at 4.00 Å. The 6-fold Zn coordination shell observed at 4.0 Å is in full accordance with Eu^{3+} being built into the hydroxide layers of the LDH structure. A similar coordination structure is observed for Europium in LDH-CO_3, Eu^{3+} being surrounded by 7 Oxygen atoms at 2.32 Å, 1 Oxygen atom at 2.84 Å (this one shorter than that in LDH-NO_3), 5(2) Oxygen atom at 3.63 Å and 6 Zn atoms at 3.93 Å. Also for LDH-CO_3 the proximity to the Zn atoms in the hydroxide layers indisputably shows Eu^{3+} to be replacing Al^{3+} in the hydroxide layers.

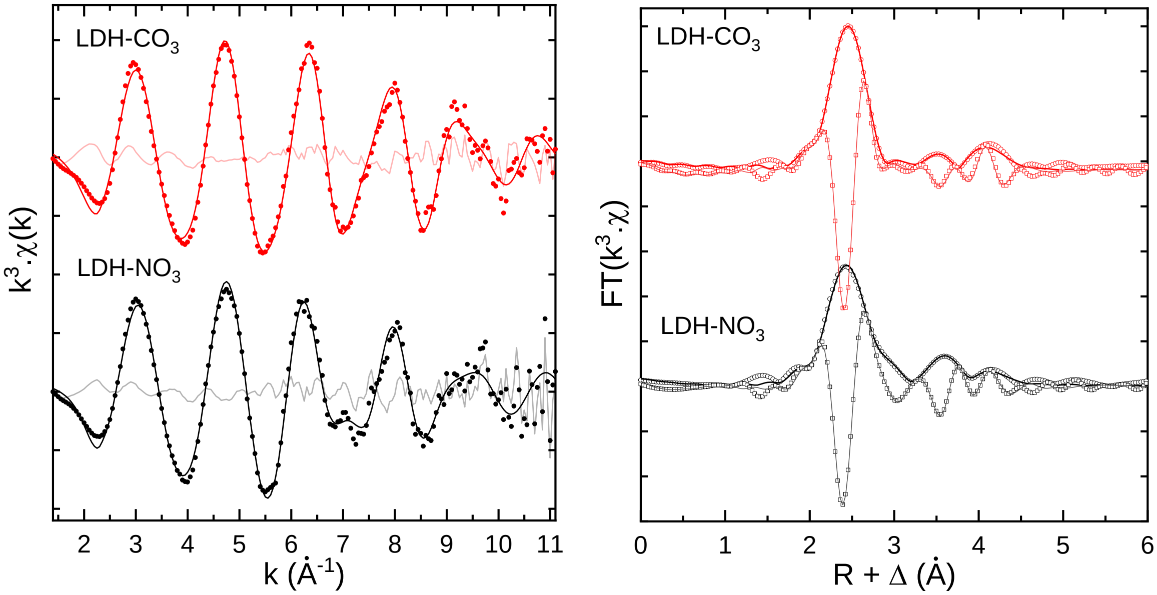

Figure S4. Eu L^{III}-edge EXAFS spectra (left) and phase-corrected Fourier transform (right) of LDH-NO_3 and LDH-CO_3. The data (circles), fits (solid lines) and residuals (faint lines) are shown with matching colors for each sample.

Table S3. Fit parameters from the Eu L^{III}-edge EXAFS data*.

Path	N^A	R (Å)^B	σ^2 (Å^2)^C
	**LDH-NO_3^{D}**		
Eu-O	7.4(3)	2.422(5)	0.0104(7)
Eu-O	1(fixed)	3.03(3)	0.013(4)
Eu-O	3.8(5)	3.569(6)	0.0083(24)
Eu-Zn	6 (fixed)	4.00(1)	0.0216(12)
	**LDH-CO_3^{E}**		
Eu-O	7.6(4)	2.434(2)	0.0088(6)
Eu-O	1(fixed)	2.84(1)	0.0037(16)
Eu-O	5(2)	3.631(3)	0.019(7)
Eu-Zn	6 (fixed)	3.93(1)	0.0178(13)

* Uncertainties are given in parentheses. An amplitude reduction factor $S_0^2 = 0.95$ was determined from a Eu_2O_3 reference. The k fitting range is 1.5-11 Å^{-1}. The R fitting range is 0.9-4.1 Å. ^{A} Coordination number. ^{B} Bond distance. ^{C} Debye-Waller factor. ^{D} Energy shift parameter $\Delta E = (3.5 \pm 0.2)$ eV, R-factor = 0.25%. ^{E} Energy shift parameter $\Delta E = (3.5 \pm 0.2)$ eV, R-factor = 0.40%.

References

1 S. J. A. Figueroa, J. C. Mauricio, J. Murari, D. B. Beniz, J. R. Piton, H. H. Slepicka, M. F. De Sousa, A. M. Espíndola and A. P. S. Levinsky, *J. Phys. Conf. Ser.*, 2016, **712**, 12022.

2 S. Marappa and P. V. Kamath, *Ind. Eng. Chem. Res.*, 2015, **54**, 11075–11079.

3 I. G. N. Silva, A. F. Morais, B. C. Lima, F. A. Garcia and D. Mustafa, *Appl. Clay Sci.*, 2020, **199**, 105861.

4 J. J. Rehr and R. C. Albers, *Rev. Mod. Phys.*, 2000, **72**, 621–654.

5 Z. K. Heiba, Y. Akin, W. Sigmund and Y. S. Hascicek, *J. Appl. Crystallogr.*, 2003, **36**, 1411–1416.

6 A. F. Morais, I. G. N. Silva, B. C. Lima, F. A. Garcia and D. Mustafa, *ACS Omega*, 2020, **5**, 23778–23785.

7 B. Ravel and M. Newville, *J. Synchrotron Radiat.*, 2005, **12**, 537–541.

8 F. Geng, H. Xin, Y. Matsushita, R. Ma, M. Tanaka, F. Izumi, N. Iyi and T. Sasaki, *Chem. - A Eur. J.*, 2008, **14**, 9255–9260.
